# Genome-Wide Role of HSF1 in Transcriptional Regulation of Desiccation Tolerance in the Anhydrobiotic Cell Line, Pv11

**DOI:** 10.3390/ijms22115798

**Published:** 2021-05-28

**Authors:** Shoko Tokumoto, Yugo Miyata, Ruslan Deviatiiarov, Takahiro G. Yamada, Yusuke Hiki, Olga Kozlova, Yuki Yoshida, Richard Cornette, Akira Funahashi, Elena Shagimardanova, Oleg Gusev, Takahiro Kikawada

**Affiliations:** 1 Department of Integrated Biosciences, Graduate School of Frontier Sciences, The University of Tokyo, Kashiwa 277-8562, Japan; 4914616910@edu.k.u-tokyo.ac.jp; 2 Division of Biotechnology, Institute of Agrobiological Sciences, National Agriculture and Food Research Organization (NARO), Tsukuba 305-0851, Japan; miyatay431@affrc.go.jp (Y.M.); cornette@affrc.go.jp (R.C.); 3 Extreme Biology Laboratory, Institute of Fundamental Medicine and Biology, Kazan Federal University, 420008 Kazan, Russia; ruselusalbus@gmail.com (R.D.); olga-sphinx@yandex.ru (O.K.); rjuka@mail.ru (E.S.); gaijin.ru@gmail.com (O.G.); 4 Department of Biosciences and Informatics, Keio University, Yokohama 223-8522, Japan; yamada@fun.bio.keio.ac.jp (T.G.Y.); hiki@fun.bio.keio.ac.jp (Y.H.); funa@bio.keio.ac.jp (A.F.); 5 Institute for Advanced Biosciences, Keio University, Tsuruoka 997-0017, Japan; feketerigoremet@gmail.com; 6 Systems Biology Program, Graduate School of Media and Governance, Keio University, Fujisawa 252-8520, Japan; 7 Laboratory for Transcriptome Technology, RIKEN Center for Integrative Medical Sciences, RIKEN, Yokohama 230-0045, Japan

**Keywords:** CRISPR/Cas9, knockout, rescue, anhydrobiosis, *Polypedilum vanderplanki*, insect cell

## Abstract

The Pv11, an insect cell line established from the midge *Polypedilum vanderplanki*, is capable of extreme hypometabolic desiccation tolerance, so-called anhydrobiosis. We previously discovered that heat shock factor 1 (HSF1) contributes to the acquisition of desiccation tolerance by Pv11 cells, but the mechanistic details have yet to be elucidated. Here, by analyzing the gene expression profiles of newly established HSF1-knockout and -rescue cell lines, we show that HSF1 has a genome-wide effect on gene regulation in Pv11. The HSF1-knockout cells exhibit a reduced desiccation survival rate, but this is completely restored in HSF1-rescue cells. By comparing mRNA profiles of the two cell lines, we reveal that HSF1 induces anhydrobiosis-related genes, especially genes encoding late embryogenesis abundant proteins and thioredoxins, but represses a group of genes involved in basal cellular processes, thus promoting an extreme hypometabolism state in the cell. In addition, HSF1 binding motifs are enriched in the promoters of anhydrobiosis-related genes and we demonstrate binding of HSF1 to these promoters by ChIP-qPCR. Thus, HSF1 directly regulates the transcription of anhydrobiosis-related genes and consequently plays a pivotal role in the induction of anhydrobiotic ability in Pv11 cells.

## 1. Introduction

Anhydrobiosis is a reversible extreme hypometabolic state characterized by almost completely stopped metabolism and extreme loss of body water, generally over 95%, due to desiccation [1,2,3]. The anhydrobiotic state enables organisms to withstand long-term drought and to return to a normal life cycle after rehydration [4]. Anhydrobiotic organisms have been found in plants and invertebrates [4,5], such as Nematoda [6], Rotifer [7], Tardigrada [8] and Insecta [9]. The larva of the sleeping chironomid *Polypedilum vanderplanki* [10], which inhabits semi-arid regions of Africa, is anhydrobiotic [11,12,13]. The cell line, Pv11, which was derived from *P. vanderplanki* embryos, is also capable of the extreme desiccation tolerance that characterizes anhydrobiosis [14,15]. Thus, Pv11 is a promising model in which to investigate the molecular mechanisms underlying anhydrobiosis in *P. vanderplanki*. Using a transcriptional approach to define the mRNA profiles of both *P. vanderplanki* larvae and Pv11 enabled us to develop a list of genes putatively involved in desiccation tolerance [16,17,18]. However, the link between the activity of these genes and the adaptation mechanisms remains unclear due to the limited range of gene manipulation techniques available in Pv11 cells.

Pv11 is the only animal cell line capable of entering anhydrobiosis [14,15], and the cells can be preserved in the dry state at room temperature for up to 372 days [9]. To successfully induce anhydrobiosis in Pv11 cells, treatment with a high concentration of trehalose is necessary prior to a desiccation step [15], and during this treatment, several genes are strongly upregulated, such as the genes encoding late embryogenesis abundant (LEA) proteins [16,19], thioredoxins (TRXs) [16,20], protein-L-isoaspartate (D-aspartate) O-methyltransferases (PIMTs) [16,21] and *Lea*-island-located (LIL) proteins [22]. LEA proteins are reported to act as a molecular shield to protect endogenous proteins from desiccation stress [23,24,25], and TRXs are a core component of the antioxidant system that attenuates oxidative stress [26]. PIMT is a protein repair enzyme that converts isoaspartate to aspartate, thus preventing distortion of the protein structure [27]. LIL genes have four to five transmembrane domains and may act as anhydro-protectants for cellular membranes, as expected from their subcellular localization [22]. Because of their function and high levels of expression, these genes are considered to be necessary for anhydrobiosis and the massive accumulation of such anhydro-protectants likely confers desiccation tolerance on Pv11 cells. However, although the above genes are considered to be important for anhydrobiosis, how they are regulated remains largely unknown.

Previously we reported that heat shock factor 1 (HSF1) is one of the pivotal transcription factors governing desiccation tolerance in Pv11 cells [17,28]. Generally, HSF1 is a master regulator of the heat shock response in mammalian cells and directly activates target genes including heat shock protein (Hsp) genes [29]. In the nematode *Caenorhabditis elegans*, HSF1 is involved both in the activation and repression of gene expression either directly or indirectly in response to heat shock [30]. In our previous study, computational analysis of the *P. vanderplanki* genome showed that a sequence similar to a HSF binding site was enriched in the promoters of desiccation- and trehalose-induced genes of *P. vanderplanki* larvae and Pv11 cells [17,28], respectively, suggesting that HSF1 regulates the expression of these genes upon desiccation stress and trehalose treatment. Transcriptome analysis of Pv11 cells is consistent with a role for HSF1 as a key transcription factor for the upregulation of anhydrobiosis-related genes [17]. However, we have yet to obtain clear evidence to determine whether HSF1 makes a substantial contribution to the upregulation of anhydrobiosis-related genes. In addition, it is unclear whether HSF1 is involved in repression of gene expression in Pv11 cells. To address these issues, a genome-wide screen for genes regulated by HSF1 is necessary, but a suitable experimental system for Pv11 cells had not been sufficiently developed prior to the current work.

To unveil the molecular mechanisms underpinning anhydrobiosis in Pv11 cells, we have developed several gene manipulation techniques, such as gene silencing and over-expression [31,32,33]. However, two problems remain unresolved: (1) electroporation is the only available method for RNA and DNA transfection in Pv11 cells, but the physical damage causes reduction of the desiccation tolerance for a while after electroporation [18]; (2) during the establishment of stable transformants, the transfected plasmid fragments could be inserted adventitiously into anhydrobiosis-related genes and thereby disrupt their function, resulting in transfected cells being significantly less desiccation-tolerant than normal Pv11 cells [18,31]. To mitigate these problems, we established a targeted gene insertion method using the CIRSPR/Cas9 system for Pv11 cells [18]. Importantly, this system can provide stable expression of exogenous genes without affecting the desiccation tolerance of Pv11 cells [18]. Such a CRISPR/Cas9-based system for gene knockout in Pv11 cells will facilitate a detailed examination of the molecular mechanisms of anhydrobiosis.

Here we describe the successful development of gene-knockout and gene-rescue methods based on the CRISPR/Cas9 system and have used these to investigate HSF1-mediated gene regulation of anhydrobiosis in Pv11 cells. Comparison of the whole-genome transcription profiles of HSF1-knockout and -rescue cell lines revealed that HSF1 is necessary for the upregulation of anhydrobiosis-related genes and the downregulation of genes involved in basal cellular processes, such as transcription and translation, during trehalose treatment. In addition, by re-expression of FLAG-tagged HSF1 in the HSF1-knockout cells, we confirmed HSF1 binding to the promoters of anhydrobiosis-related genes by ChIP-qPCR using a FLAG antibody. This result suggests that HSF1 can directly activate genes during trehalose treatment in Pv11 cells. Moreover, the expression of *Lea* and *Trx* genes was highly dependent on the presence of HSF1—to a greater extent than other genes—showing that HSF1 is the main activator of *Lea* and *Trx* genes in Pv11 cells. Our results demonstrate that, for successful entry of Pv11 cells into anhydrobiosis, genome-wide transcriptional regulation by HSF1 is required.

## 2. Results

### 2.1. Establishment of a HSF1-Knockout Clonal Cell Line Using the CRIS-PITCh Technique

**Figure 1:**
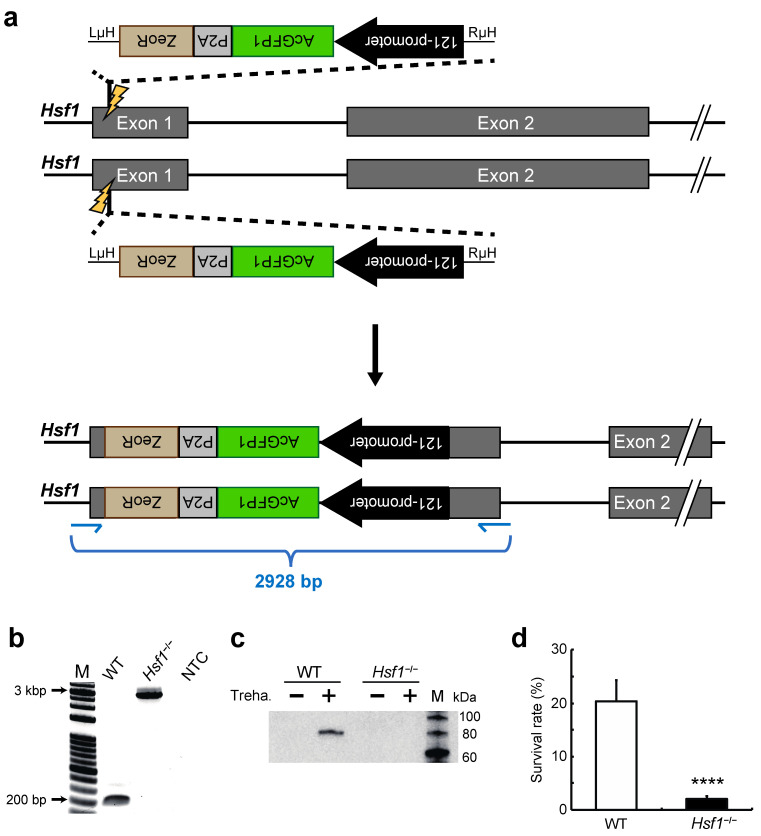
Establishment of an HSF1-knockout (*Hsf1*^−/−^) cell line: (**a**) Schematic diagram of *Hsf1* knockout in Pv11 cells using the CRIS-PITCh (CRISPR/Cas9-mediated precise integration into target chromosome) system. The donor vector harbors 121-promoter-AcGFP1-P2A-ZeoR sequences flanked by microhomology arms and was transfected with gRNA- and hSpCas9-expression vectors into Pv11 cells, resulting in insertion of the AcGFP1 and ZeoR expression units into exon 1 of *Hsf1*. The thin blue arrows show the primer binding sites for the genomic PCR shown in (**b**). The primer sequences are given in Data S1. LμH and RμH, left and right microhomology, respectively; (**b**) PCR analysis of ZeoR^+^ and AcGFP1^+^ clonal cell line. The product sizes of the WT and the clonal cell line are 191 bp and 2928 bp, respectively. The sequence is given in Data S1. WT, wild type Pv11 cells; M, molecular size markers; NTC, no-template control; (**c**) Western blotting analysis of the *Hsf1*^−/−^ clonal cell line using HSF1 antibody. The same membrane stained with ponceau S to validate protein transfer is shown in Figure S2. Treha., treatment for 48 h with trehalose mixture; (**d**) survival rate after desiccation-rehydration of the *Hsf1*^−/−^ clonal cell line. The number of live cells was counted one day after rehydration. Normalized values are expressed as mean ± standard deviation (SD). **** *p* < 0.0001; n = 6 in each group.

**Figure 2:**
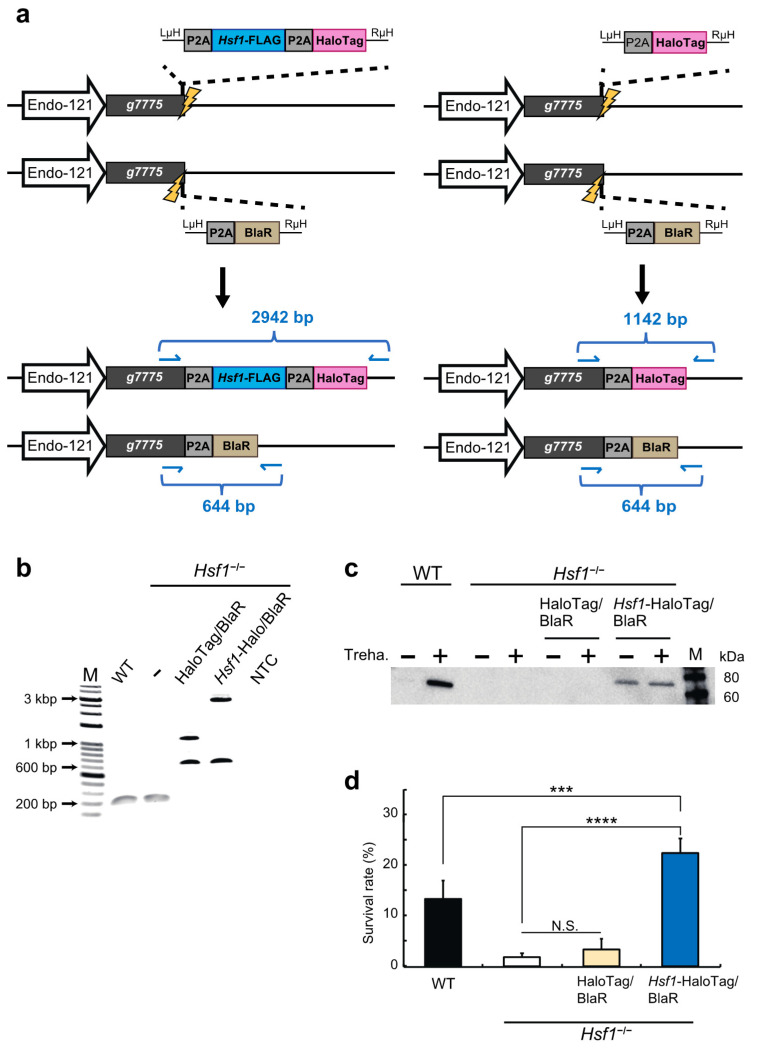
Establishment of an HSF1-rescue cell line: (**a**) Schematic diagram of the knock-in of either *Hsf1*-FLAG-P2A-HaloTag plus BlaR or HaloTag alone plus BlaR into *Hsf1*^−/−^ cells using the CRIS-PITCh system. These genes were knocked into the 5′ flanking site of the stop codon of the *g7775* gene. The HaloTag-plus-BlaR knock-in cell line was established as a negative control for the HSF1-rescue cell line. The thin blue arrows show the primer binding sites for genomic PCR. The primer sequences are given in Data S2 and S3. Endo-121, endogenous 121-promoter; LμH and RμH, left and right microhomology, respectively; (**b**) PCR analysis of *Hsf1*^−/−^; HaloTag/BlaR and *Hsf1*^−/−^; *Hsf1*-HaloTag/BlaR cell lines. The product sizes of WT, *Hsf1*^−/−^, HaloTag, BlaR and *Hsf1*-FLAG-P2A-HaloTag are 181 bp, 1142 bp, 644 bp and 2942 bp, respectively. The respective genome sequences are given in Data S2 and S3. M, molecular size markers; WT, wild type Pv11 cells; NTC, no-template control; (**c**) Western blotting analysis of *Hsf1*^−/−^; HaloTag/BlaR and *Hsf1*^−/−^; *Hsf1*-HaloTag/BlaR cells using HSF1 antibody. The same membrane stained with ponceau S to validate protein transfer is shown in Figure S2; (**d**) survival rate after desiccation-rehydration of *Hsf1*^−/−^; HaloTag/BlaR and *Hsf1*^−/−^; *Hsf1*-HaloTag/BlaR cells. The number of live cells was counted one day after rehydration. Normalized values are expressed as mean ± SD. *** *p* < 0.001; **** *p* < 0.0001; N.S., not significant; n = 5 in each group.

To establish a HSF1-knockout cell line using the CRISPR/Cas9-mediated PITCh (precise integration into target chromosome) approach, termed a CRIS-PITCh system [34], we designed the following vectors to integrate AcGFP1 and zeocin resistance gene (ZeoR) with opposite orientation in the *Hsf1* gene [35]: (1) an expression vector for gRNA targeting exon 1 of *Hsf1* (Figure 1a); (2) a donor vector harboring a 121-promoter-AcGFP1-P2A-ZeoR flanked by microhomology arms and the gRNA sequence. The donor vector plus hSpCas9- [18] and gRNA-expression vectors were transfected into Pv11 cells and we then performed zeocin selection and single-cell sorting as described previously [18] to acquire a *Hsf1*-disrupted clonal cell line of Pv11 cells, *Hsf1*^−/−^ (Figure 1a).

To confirm that the gene insertion had occurred correctly, we extracted genomic DNA from the clonal cell line and performed genomic PCR on the region around the inserted gene. Whereas a single 2928-bp band was detected in the *Hsf1*^−/−^ cell line, a single 191-bp band was detected in wild type (WT) Pv11 cells (Figure 1b). This result showed biallelic insertion of the 121-promoter-AcGFP1-P2A-ZeoR unit into exon 1 of *Hsf1* in the *Hsf1*^−/−^ cell line. Furthermore, analysis of the genome sequence of *Hsf1* confirmed correct gene knock-in (Data S1). Western blotting using a HSF1-specific antibody confirmed the absence of HSF1 protein both before (T0) and 48 h after treatment with a high concentration of trehalose (T48, Figure S1) in the *Hsf1*^−/−^ cell line (Figure 1c and Figure S2a). These results clearly demonstrate the successful establishment of a HSF1-knockout cell line. The survival rate of the dried *Hsf1*^−/−^ cell line after rehydration was significantly lower than that of dried WT cells (Figure 1d), shows that HSF1 is important for desiccation tolerance in Pv11, as described in our previous report [28].

### 2.2. HSF1 Rescue of the HSF1-Knockout Cell Line Using the CRIS-PITCh Technique

To create an HSF1-rescue cell line from the *Hsf1*^−/−^ cell line, constructs encoding *Hsf1*-Flag-P2A-HaloTag and blasticidin S-resistance (BlaR) were introduced into the 5′-flanking site of stop codon of *g7775* (previously called *Pv.00443*) [18], as illustrated in the left panel of Figure 2a. In a separate experiment, HaloTag alone and BlaR gene were also integrated into the same locus to generate a negative control for the rescue cell line (Figure 2a right). The donor vectors plus hSpCas9- and gRNA-expression vectors were transfected into the *Hsf1*^−/−^ cell line, then blasticidin S selection and single-cell sorting were performed as described previously [18].

The genomic DNAs of the clonal cell lines were subjected to genomic PCR and sequenced to confirm precise insertion of the above constructs. In the HaloTag and BlaR knock-in cell line, 1142 and 644 bp bands were detected, while 2942 and 644 bp bands were detected in the *Hsf1*-Flag-P2A-HaloTag and BlaR knock-in cell line (Figure 2b, sequence data are shown in Data S2 and S3). These results confirmed the establishment of the *Hsf1*^−/−^; *g7775*^HaloTag/BlaR^ (hereinafter called “*Hsf1*^−/−^; HaloTag/BlaR”) and *Hsf1*^−/−^; *g7775^Hsf1^*^-HaloTag/BlaR^ (hereinafter called “*Hsf1*^−/−^; *Hsf1*-HaloTag/BlaR”) cell lines. Western blotting showed the re-expression of HSF1 protein only in the *Hsf1*^−/−^; *Hsf1*-HaloTag/BlaR cell line (Figure 2c and Figure S2b). Furthermore, the desiccation survival rate was completely rescued only in the *Hsf1*^−/−^; *Hsf1*-HaloTag/BlaR cell line (Figure 2d), that is, in the HSF1-rescue cell line. Intriguingly, the loss of HSF1 did not affect cell viability upon treatment with a high concentration of trehalose for 48 h, which induces desiccation tolerance in Pv11 cells (Figure S3). This suggests that HSF1 is likely involved in regulating the expression of genes that enable Pv11 cells to withstand the stress of drying and rehydration, rather than the physical stresses, such as osmotic stress, caused by high concentrations of trehalose per se [15,18].

### 2.3. Contribution of HSF1 to the Regulation of Genes Crucial for Anhydrobiosis

To examine the contribution of HSF1 to the regulation of genes involved in desiccation tolerance, total mRNA was extracted from WT, *Hsf1*^−/−^, *Hsf1*^−/−^; HaloTag/BlaR and *Hsf1*^−/−^; *Hsf1*-HaloTag/BlaR cells at T0 and T48, then subjected to transcriptome analysis. The result of principal component analysis is shown in Figure S4a: two conditions in *Hsf1*^−/−^ and *Hsf1*^−/−^; HaloTag/BlaR cells clustered together, and a large difference was observed between WT and *Hsf1*^−/−^; *Hsf1*-HaloTag/BlaR cells in both conditions. These data indicated that the two HSF1-knockout cells have a similar expression pattern, while the expression patterns of WT and *Hsf1*^−/−^; *Hsf1*-HaloTag/BlaR cells are different, probably due to HSF1 expression at T0 in *Hsf1*^−/−^; *Hsf1*-HaloTag/BlaR cells (Figure 2c).

Next, we compared mRNA expression for the T0 and T48 conditions in the four cell lines. As a result, 7636 DEGs were identified (edgeR: likelihood ratio test, false discovery rate (FDR) < 0.05; Table S1) and clustered using a hierarchical clustering algorithm (Figure S4b and Data S4). Most genes in cluster A showed the highest expression level in *Hsf1*^−/−^; *Hsf1*-HaloTag/BlaR cells treated with trehalose (Figure S4b,c), and the cluster includes many known anhydrobiosis-related genes such as *Lea* and *Pimt* genes [16,19,21] (Table S2). In the *Hsf1*^−/−^; *Hsf1*-HaloTag/BlaR cells, the high expression level of cluster A genes may be the reason for the statistically significant increase in the survival rate of the dried cells after rehydration (Figure 2d and Figure S4b).

**Figure 3:**
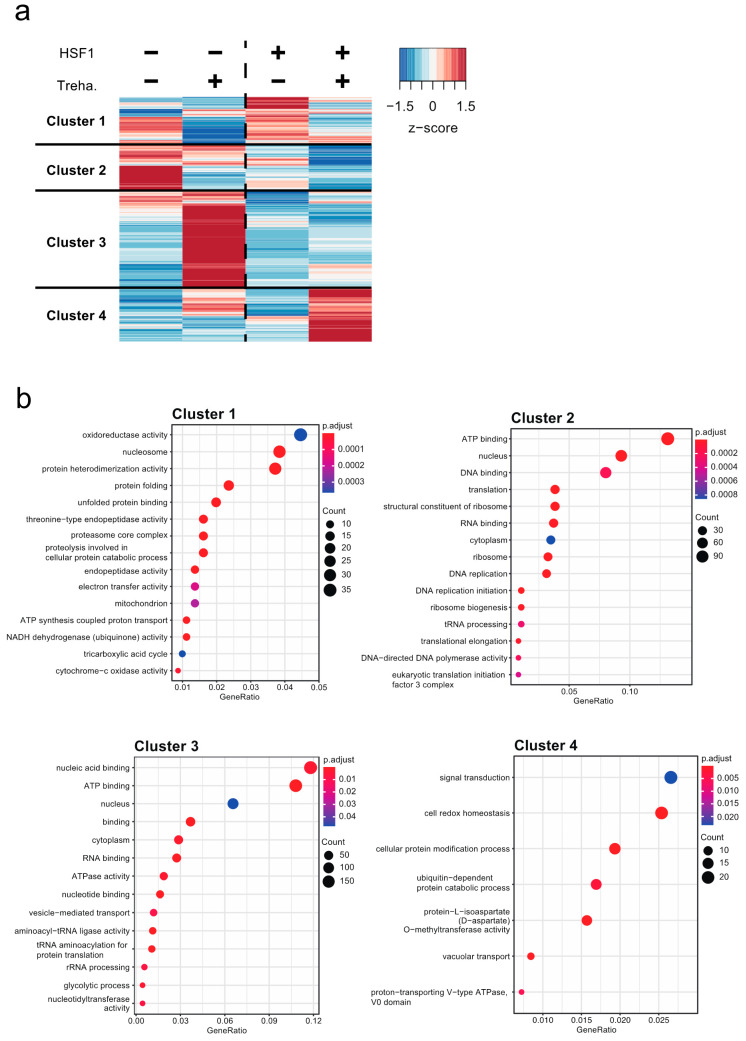
Transcriptome changes in Pv11 cells following HSF1-knockout and -rescue: (**a**) Hierarchical clustering based on TPM of *Hsf1*^−/−^; HaloTag/BlaR and *Hsf1*^−/−^; *Hsf1*-HaloTag/BlaR cell lines during trehalose treatment. Red and blue color indicates high expression level and low expression level, respectively. The horizontal and vertical axes show sample and the number of differentially expressed genes (DEGs), respectively. HSF1−, *Hsf1*^−/−^; HaloTag/BlaR; HSF1+, *Hsf1*^−/−^; *Hsf1*-HaloTag/BlaR; Treha. −, before trehalose treatment (T0); Treha. +, after trehalose treatment (T48); (**b**) GO enrichment analysis for each cluster in (**a**). All genes with or without GO annotation are listed in Data S4.

To focus on gene regulation by HSF1, we compared the mRNA expression profiles of *Hsf1*^−/−^; HaloTag/BlaR and *Hsf1*^−/−^; *Hsf1*-HaloTag/BlaR cells for the following reasons: (1) the two cell lines were derived from a common cell line, *Hsf1*^−/−^; (2) the only difference between the two cell lines is the expression of HSF1, because AcGFP1, ZeoR, HaloTag and BlaR are expressed in both cell lines. This analysis identified 5930 DEGs (edgeR: likelihood ratio test, FDR < 0.05; Table S3). All DEGs were clustered using a hierarchical clustering algorithm, and then Gene Ontology (GO) enrichment analysis was performed for each cluster (Figure 3 and Data S5).

In cluster 1, which comprised 1140 DEGs (Figure 3a and Table S3), most genes were downregulated at T48 in both cell lines (Figure 3a and Figure S5), while at T0, the expression levels of this cluster tended to be higher in the presence of HSF1 (Figure S5 and Figure 2c). These results suggested that this cluster contains a group of HSF1-inducible genes at T0. In this cluster, the GO terms, oxidoreductase activity (GO:0016491), nucleosome (GO:0000786) and protein heterodimerization activity (GO:0046982) were enriched (Figure 3b and Data S5). Genes encoding alcohol-, aldehyde-, or lactate/malate-dehydrogenase domains were included in the oxidoreductase activity (Data S5), and genes encoding histone domains were included in the nucleosome and protein heterodimerization activity (Data S5). In addition, genes related to chaperones including *Hsp* genes were included in the protein folding (GO:0006457) and unfolded protein binding (GO:0051082, Data S5) categories, and these GOs were only enriched in this cluster (Figure 3b). This result showed that several *Hsp* genes are downregulated during trehalose treatment.

There were 1132 DEGs in cluster 2 (Figure 3a and Table S3). These genes were generally downregulated at T48 in the presence of HSF1 and had a higher expression level at T0 or T48 in the absence of HSF1 (Figure 3a and Figure S5), suggesting that this cluster contains a group of HSF1- and trehalose-repressed genes. In this cluster, the GO terms, ATP binding (GO:0005524), nucleus (GO:0005524) and DNA binding (GO:0003677) were enriched (Figure 3b). Genes encoding helicase, protein kinase or AAA+ ATPase domains were included in the ATP binding category, and genes encoding histone and transcription factor-like genes were included in the nucleus and DNA binding group (Data S5). Furthermore, genes relating to translation were also enriched in this cluster (e.g., translation: 0006412 and structural consistent of ribosome: 0003735). Genes relating to protein kinase may be involved in signaling pathways regulated by phosphorylation [36]. The AAA+ ATPase family is involved in a myriad of cellular processes, for example, membrane fusion, protein folding/unfolding and protein degradation [37,38,39,40]. Therefore, these data suggested that HSF1 may repress genes involved in basal cellular processes including signaling pathways, transcription and translation.

In cluster 3, there were 2338 DEGs (Figure 3a and Table S3), almost all of which were strongly upregulated at T48, but only in the absence of HSF1 (Figure 3a and Figure S5), suggesting that this cluster contains a group of HSF1-repressed and trehalose-inducible genes. In this cluster, the GO terms nucleic acid binding (GO:0003676), ATP binding (GO:0005524) and nucleus (GO:0005524) were enriched (Figure 3b). Many genes encoding zinc finger domains were included in the nucleic acid binding and nucleus category, while genes encoding AAA+ ATPase, ABC transporter-like, protein kinase or helicase domain were included in the ATP binding group (Data S5). The genes encoding zinc finger domains are probably transcription factors and are thus involved in transcription [41,42]. ABC transporters are ATP-dependent pumps that transport a huge diversity of substrates and are thus involved in a wide range of cellular processes [43,44]. Therefore, these results suggested that cluster 3 genes, which are involved in many cell processes including transcription, signal transduction and the transportation of substrates, are repressed by HSF1 during trehalose treatment. 

Cluster 4 comprised 1320 DEGs, and many anhydrobiosis-related genes were included in this cluster (Table S4). Most of the cluster 4 genes were highly expressed at T48 only in the presence of HSF1 (Figure 3a and Figure S5), showing that genes in this cluster were upregulated in an HSF1- and trehalose-codependent manner (Figure 3a and Figure S5). In this cluster, the GO terms signal transduction (GO:0007165), cell redox homeostasis (GO:0045454) and cellular protein modification process (GO:0006464) were enriched (Figure 3b and Data S5). These results suggested that massive accumulation of proteins in this cluster could contribute to tolerance of desiccation and rehydration stresses in Pv11 cells.

There were also genes that are up- or downregulated regardless of the presence or absence of HSF1; however, there were fewer of these genes than of the DEGs in Figure 3 (Table S3); 606 and 504 genes were up- and downregulated, respectively, both in *Hsf1*^−/−^; HaloTag/BlaR and *Hsf1*^−/−^; *Hsf1*-HaloTag/BlaR cells (Data S6, see the supplementary methods for detailed calculation). The most enriched GO term among the upregulated genes was signal transduction (GO:0007165; Figure S6 and Data S7). Among the downregulated genes, ribosomal protein-related GOs (structural consistent of ribosome: 0003735, ribosome: 0005840 and translation: 0006412) were enriched (Figure S6 and Data S7). These results showed that the activation of signaling pathways and the repression of translation also occurred in an HSF1-independent manner.

### 2.4. Direct Transcriptional Regulation of Cluster 4 Genes by HSF1

**Figure 4:**
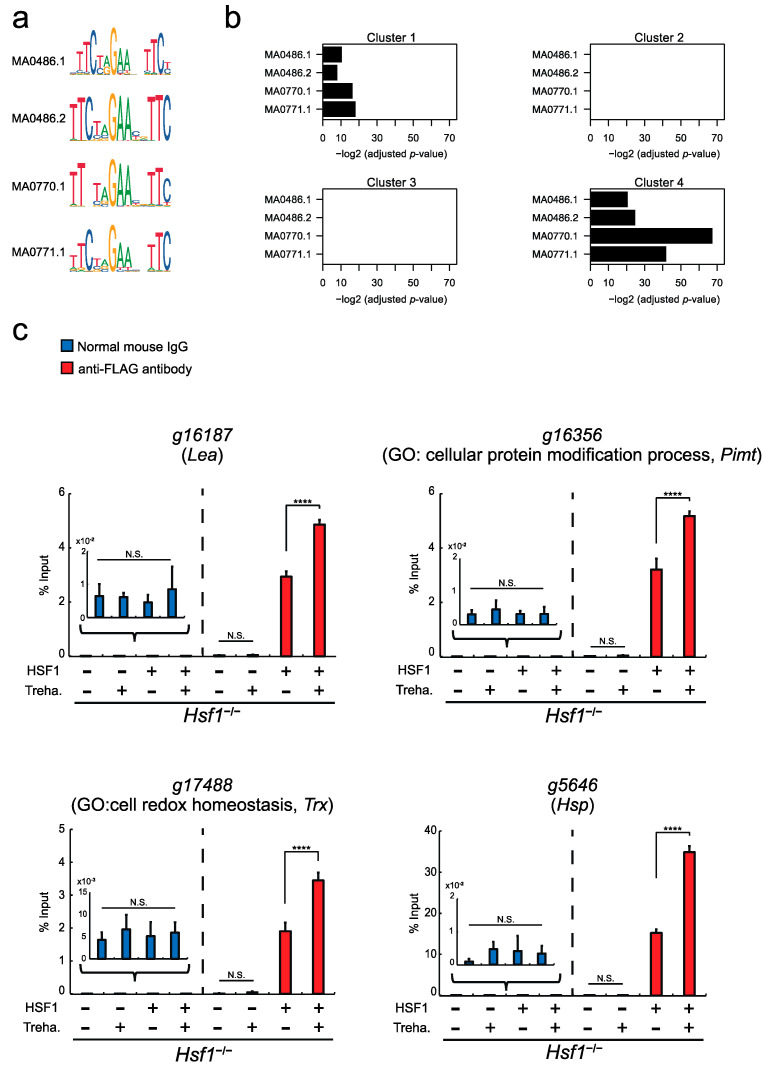
Heat shock element (HSE) enrichment analysis in gene promoters and confirmation of HSF1 binding: (**a**) HSF binding sequences that are registered as HSEs in the JASPAR2018 CORE database; (**b**) HSE enrichment analysis in each cluster of Figure 3a. It is predicted that HSEs are most enriched in the promoter regions of cluster 4 genes, as defined in Figure 3a; (**c**) ChIP assay of HSF1 binding to the promoters of four cluster 4 genes, performed on *Hsf1*^−/−^; HaloTag/BlaR and *Hsf1*^−/−^; *Hsf1*-HaloTag/BlaR cell lines. FLAG antibody was used to immunoprecipitate HSF1-FLAG/DNA complexes, and normal mouse IgG was used as a negative control. Normalized values are expressed as mean ± SD. Treha. −, before trehalose treatment (T0); Treha. (+), after trehalose treatment (T48); HSF1 (−); *Hsf1*^−/−^; HaloTag/BlaR; HSF1+, *Hsf1*^−/−^; *Hsf1*-HaloTag/BlaR; **** *p* < 0.0001; N.S., not significant; n = 3 in each group.

To examine the transcriptional activity of HSF1 via a specific promoter site, called a heat shock element (HSE) [29,45], in Pv11 cells, we performed a luciferase assay in *Hsf1*^−/−^ cells using a reporter vector harboring a canonical HSE (Figure 4a: MA0486.1) either with or without vector-driven *Hsf1* expression. As shown in Figure S7, luciferase activity was strongly induced only when *Hsf1* was also expressed. This suggests that *P. vanderplanki* HSF1 can bind the canonical HSE sequence and promote the transcription of downstream genes. 

Next, to examine whether the genes in Figure 3 were regulated by HSF1 directly, we analyzed the respective promoter regions (−500/+500 bp from TSS) for the presence of HSEs (Data S8). As shown in Figure 4b, HSEs were most enriched in the promoters of cluster 4 genes (Fisher’s exact test, adjusted *p*-value < 5.75 × 10^−21^; Figure 4b and Table S5), suggesting that HSF1 is likely to directly regulate the genes in cluster 4. To confirm the binding of HSF1 to the promoter regions of these genes, four genes with HSEs in their promoters were randomly selected from Data S8. ChIP-qPCR was performed, using an anti-FLAG antibody for the ChIP experiment and normal IgG as a negative control, and revealed HSF1 binding to the promoters of all four genes in *Hsf1*^−/−^; *Hsf1*-HaloTag/BlaR cells (Figure 4c). Furthermore, the binding of HSF1 to the promoters was increased by trehalose treatment (Figure 4c), which is congruent with the expression pattern of cluster 4 genes (Figure 3a). These data suggest that HSF1 can bind to the promoters of cluster 4 genes and thereby directly activate gene expression in Pv11 cells.

### 2.5. Differential Contribution of HSF1 to the Expression of Anhydrobiosis-Related Genes

**Figure 5:**
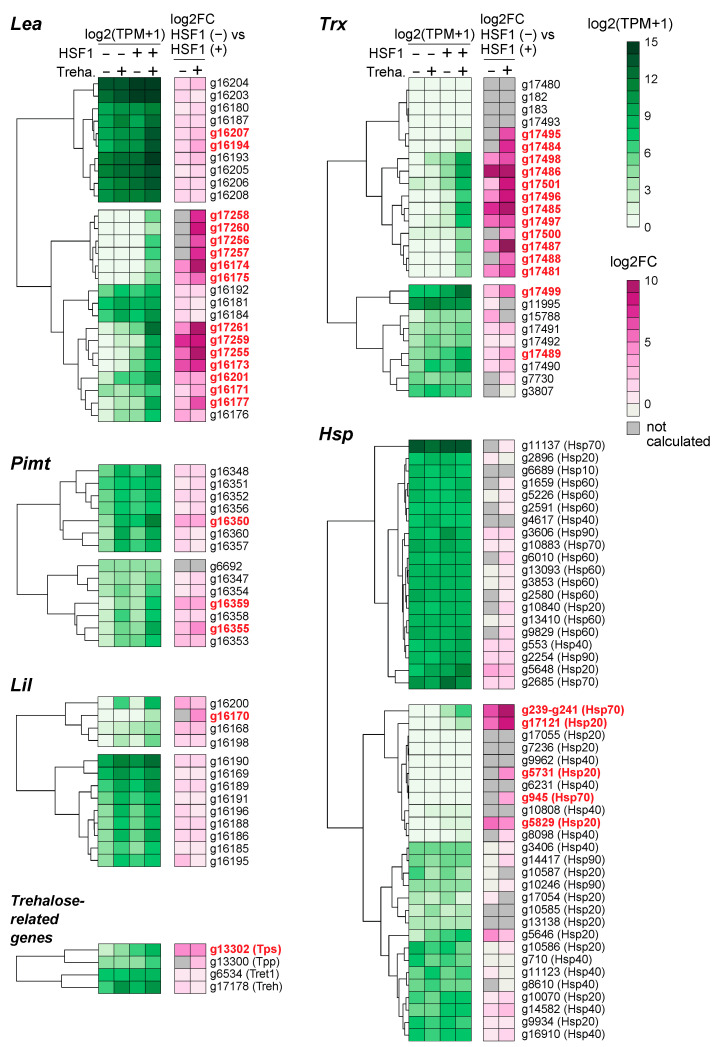
Heatmaps of mRNA-seq log2 (TPM: tags per kilobase per million + 1) and log2FC values for the anhydrobiosis-related genes *Lea*, *Trx*, *Lil*, *Pimt*, *Hsp* and trehalose metabolism-related genes. The log2 (TPM +1) values were calculated by applying log2 on TPM + 1 values. Fold-changes were calculated by comparing TPM values of *Hsf1*^−/−^; *Hsf1*-HaloTag/BlaR and *Hsf1*^−/−^; HaloTag/BlaR cell lines before (T0) or after trehalose treatment (T48) by using edgeR. All log2 (TPM+1) and log2FC values are shown in Data S10. Red gene names in bold highlight changes in gene expression where log2FC ≥ 3 (TPM comparison, HSF (−) vs. HSF (+) in T48). HSF1 (−), *Hsf1*^−/−^; HaloTag/BlaR; HSF1 (+), *Hsf1*^−/−^; *Hsf1*-HaloTag/BlaR; Treha. −, before trehalose treatment (T0); Treha. +, after trehalose treatment (T48); not calculated; the log2FC values were not calculated when the both TPM values were zero.

Most anhydrobiosis-related genes [13,16,22,28,46,47] belong to cluster 4 and are upregulated in an HSF1- and trehalose-codependent manner (Table S4 and Figure 3). To investigate the contribution of HSF1 to the expression of anhydrobiosis-related genes, the TPM values of *Lea*, *Trx*, *Pimt*, *Lil*, *Hsp* (Data S9) and trehalose metabolism-related genes were compared in *Hsf1*^−/−^; *Hsf1*-HaloTag/BlaR and *Hsf1*^−/−^; HaloTag/BlaR cells. As shown in Figure 5, approximately 60% (83 out of 130) of the anhydrobiosis-related genes were more highly expressed at T48 in *Hsf1*^−/−^; *Hsf1*-HaloTag/BlaR cells than in *Hsf1*^−/−^; HaloTag/BlaR cells (log2FC ≥ 1; Data S10). This result showed that HSF1 contributes to the induction of most anhydrobiosis-related genes in Pv11 cells. In addition, 39 of the 83 genes were strongly upregulated in an HSF1- and trehalose-codependent manner (log2FC ≥ 3; Figure 5 and Data S10). Fifteen and 14 of these 39 genes were *Lea* and *Trx* genes, respectively. On the other hand, only 3, 1, 5 and 1 of the *Pimt*, *Lil*, *Hsp* and trehalose metabolism-related genes, respectively, were strongly upregulated (log2FC ≥ 3; Figure 5 and Data S10). These results showed that the expression of *Lea* and *Trx* genes is more dependent on HSF1 than that of *Pimt*, *Lil*, *Hsp* and trehalose metabolism-related genes in Pv11 cells.

## 3. Discussion

The first and still the only anhydrobiotic animal cell line, Pv11, was reported in 2010 [14]. Since then, practical and effective genetic manipulation techniques for this cell line have gradually been developed [18]. In this study, we demonstrate the successful establishment of HSF1-knockout (*Hsf1*^−/−^ and *Hsf1*^−/−^; HaloTag/BlaR) and -rescue (*Hsf1*^−/−^; *Hsf1*-HaloTag/BlaR) cell lines using the CRIS-PITCh system [34,35] (Figure 1 and Figure 2) and investigate the contribution of HSF1 to the gene regulation required for anhydrobiosis in Pv11 cells (Figure 3). Comparison of the expression profiles of *Hsf1*^−/−^; HaloTag/BlaR and *Hsf1*^−/−^; *Hsf1*-HaloTag/BlaR cells showed that HSF1 is involved in the induction and repression of genes in response to trehalose treatment. In addition, most anhydrobiosis-related genes are upregulated in an HSF1- and trehalose-codependent manner (Figure 5). Moreover, HSF1 binding to the promoters of anhydrobiosis-related genes was confirmed by the re-expression of FLAG-tagged HSF1 in *Hsf1*^−/−^ cells (Figure 4). These results indicate that HSF1 directly induces anhydrobiosis-related genes, in particular *Lea* and *Trx* genes. To the best of our knowledge, this is the first report of the successful construction of a gene-knockout and -rescue system in a non-model anhydrobiotic animal. 

The establishment of an HSF1-knockout cell line, whose anhydrobiotic capability is severely compromised, involved successful integration of the AcGFP1/ZeoR expression unit in *Hsf1* exon 1 (Figure 1). In confirmation of the important role played by HSF1, the anhydrobiotic potential of the knockout line was restored in an HSF1-rescue cell line (Figure 2). Our results clearly demonstrate that the CRISPR/Cas9-mediated knockin/knockout method is efficient in Pv11 cells. This technique will facilitate clarification of gene regulation by other transcription factors, for example, NF-YC, NFAT and CREB, which are reported to potentially contribute to desiccation tolerance in Pv11 cells [17,18]. In addition, the ChIP experiment targeting FLAG-tagged HSF1 carried out in Pv11 cells (Figure 4c), shows that ChIP can be performed by re-expressing FLAG-tagged factors of interest in Pv11 cells [48,49,50]. Unlike in model organisms, specific antibodies are often not available in non-model organisms, so that generating a specific ChIP-grade antibody is a major obstacle to performing ChIP experiments [51]. Together, these technical advances provide a basic toolkit for uncovering the molecular mechanisms underlying anhydrobiosis. In forthcoming research, the establishment of knockout and rescue cell lines of target genes will help to define the anhydrobiosis transcriptional regulatory network [17]. 

HSF1 was recently reported as a regulatory factor of anhydrobiosis in *P. vanderplanki* [28] and *Artemia franciscana* [52], and HSF1 is considered to be an activator of anhydrobiosis-related genes, such as *Trx* and *Hsp* genes [28,52,53]. However, only a few genes had previously been described as HSF1-regulated [28,52], and it was not clear whether HSF1 makes a substantial contribution to the regulation of anhydrobiosis-related genes. In the current study, we showed that not only was HSF1 involved in the activation of cluster 4 genes, which include anhydrobiosis-related genes, but also in repressing the expression of cluster 2 and 3 genes, which are involved in cellular processes such as signal transduction, transcription and translation (Figure 3 and Data S5). These data demonstrate that HSF1 works as both activator and repressor in Pv11 cells. The repression of cellular process genes is thought also to be important for entering anhydrobiosis in *P. vanderplanki*, because many metabolic and cellular processes that operate under normal conditions come to a halt in *P. vanderplanki* larvae and Pv11 during desiccation and trehalose treatment [28,47,54]. Although the repression mechanism for such cellular processes has not been yet described, our current results suggest only indirect regulation of the respective genes by HSF1, because HSEs were not enriched in their promoters (Figure 4b). Probably HSF1 represses these genes via other transcription factors [17]. Furthermore, our results clearly showed that HSF1 is involved in the induction of thousands of genes, including anhydrobiosis-related genes (Figure 3 and Figure 5), and support the notion of direct regulation of anhydrobiosis-related genes by HSF1 (Figure 4). To investigate the detailed mechanisms of regulation by HSF1 and to identify genome-wide HSF1 binding sites, ChIP-seq will be performed in our forthcoming study.

We revealed that the expression of most *Lea* and *Trx* genes is highly dependent on the presence of HSF1 during trehalose treatment (Figure 5), showing that HSF1 is a major activator of the gene transcription. In contrast, most *Pimt*, *Lil* and *Hsp* genes show a lower dependency on HSF1 for their expression during trehalose treatment (Figure 5 and Data S10). This result indicates the existence of other factors that strongly regulate the *Pimt*, *Lil* and *Hsp* genes following trehalose treatment. Indeed, we have shown that several transcription factors may contribute to the acquisition of desiccation tolerance by Pv11 cells via a complex regulatory system [17,18]. To efficiently catalogue these candidate transcription factor genes, several genome-wide screening methods may be useful, for example, massively parallel reporter assays [55,56] and CRISPR screening [57,58]. We have already established the basic technology for these screening systems [18,33]. Therefore, combining these advances should further identify genes critical for anhydrobiosis in Pv11 cells.

## 4. Materials and Methods

### 4.1. Cell Culture

Pv11 cells were originally established in our laboratory [14]. Pv11 cells and all clonal cell lines were grown in IPL-41 medium (Thermo Fisher Scientific, Waltham, MA, USA) supplemented with 2.6 g/L tryptone phosphate broth (Becton, Dickinson and Company, Franklin Lakes, NJ), 10% (*v*/*v*) fetal bovine serum (MP Biomedicals, Santa Ana, CA, USA), and 0.05% (*v*/*v*) of an antibiotic and antimycotic mixture (penicillin, amphotericin B, and streptomycin; Merck KGaA, Darmstadt, Germany).

### 4.2. Vector Construction

gRNA was designed as described previously [18]. To construct gRNA expression vectors, pPvU6b-DmtRNA-BbsI [18] was digested with BbsI and the digested small fragment was replaced with the gRNA sequence generated by annealing the following oligonucleotides: sense: 5′-TGCAATAATTTTGCCAAGAATGCA-3′; antisense: 5′-AAACTGCATTCTTGGCAAAATTAT-3′ (Data S11). To construct the donor vector for establishing the *Hsf1*^−/−^ cell line, first, a 121-promoter-AcGFP1-P2A-ZeoR expression vector was constructed. AcGFP1 and P2A-ZeoR were amplified using specific primers (Table S6: set 1 and 2), then assembled with pP121K-AcGFP1 [32] digested with BamHI and SacII. Next, to add the microhomology and gRNA sequences, the gRNA-LμH-121-AcGFP1-P2A-ZeoR-RμH-gRNA sequence was amplified using specific primers (Table S6: set 3) and inserted into the pCR™-Blunt II-TOPO^®^-vector (Thermo Fisher Scientific, pCR-*Hsf1*gRNAµH-121-AcGFP1-P2A-ZeoR; Data S12).

To construct the donor vectors for establishing the *Hsf1*^−/−^; HaloTag/BlaR and *Hsf1*^−/−^; *Hsf1*-HaloTag/BlaR cell lines, pCR4-*g7775*gRNAµH-P2A-*Hsf1*-3xFLAG-P2A-HaloTag and pCR4-*g7775*gRNAµH-P2A-BlaR were designed. First, *Hsf1* cDNA was cloned from cDNA of dried *P. vanderplanki* larvae using specific primers and sequenced (Table S6: set 4). Then, to add the 3xFLAG sequence, pPv121*-Hsf1*-3xFLAG was constructed in the pPv121-MCS vector [33] using NEBuilder HiFi DNA Assembly Mater Mix (New England BioLabs, Ipswich, MA, USA; Table S6: set 5). The pPv121*-Hsf1*-3xFLAG expression vector and the pCR4- *g7775*gRNAµH-P2A-HaloTag donor vector [32] were used as a PCR template to construct the pCR4-*g7775*gRNAµH-P2A-*Hsf1*-3xFLAG-P2A-HaloTag donor vector. PCR was performed using specific primers (Table S6: set 6 and 7). These PCR products were inserted into pCR4-*g7775*gRNAµH-P2A-BbsI [32] digested with BbsI using NEBuilder HiFi DNA Assembly Mater Mix (pCR4-*g7775*gRNAµH-P2A-*Hsf1*-3xFLAG-P2A-HaloTag; Data S13). For construction of the pCR4-*g7775*gRNAµH-P2A-BlaR donor vector, the BlaR gene was cloned using specific primers (Table S6: set 8) from the pYES6/CT (Thermo Fisher Scientific) and inserted into pCR4-*g7775*gRNAµH-P2A-BbsI [32] digested with BbsI using NEBuilder HiFi DNA Assembly Mater Mix (pCR4-*g7775*gRNAµH-P2A- BlaR; Data S14).

### 4.3. Transfection and Cell Sorting 

The cells used in each experiment were seeded at a density of 3 × 10^5^ cells per mL into a 25 cm^2^ cell culture flask and grown at 25 °C for 4–6 days before transfection. Transfection into Pv11 cells was carried out using a NEPA21 Super Electroporator (Nepa Gene, Ichikawa, Chiba, Japan) as described previously [31]. Five μg each of the gRNA- (previously constructed and constructed above) and hSpCas9-expression [18] vectors and 0.03–0.1 pmol donor vectors constructed above were transfected into cells. The combination of transfected vectors is shown in Table S7. 

For establishment of the HSF1-knockout cell line in Figure 1, the donor vector harboring AcGFP1 and ZeoR expression cassettes were integrated into the Exon1 of *Hsf1* with the opposite orientation in the exogenous *Hsf1* [35]. For establishment of the HSF1-rescue cell line in Figure 2, the donor vector harboring *Hsf1* was integrated into the 5′-flanking site of stop codon of *g7775* because it allows exogenous gene expression without loss of desiccation tolerance in Pv11 cells [18]. 

Five days after transfection, cells were treated with 400 µg/mL zeocin or 200 µg/mL blasticidin at a density of 1 × 10^5^ cells per mL. One week after this treatment, the medium was changed to normal IPL-41 medium, and the cells were grown for a further two weeks. Single-cell and bulk sorting were performed by selecting for AcGFP1 or HaloTag fluorescence as described previously [18]. 

### 4.4. Genomic PCR and Western Blotting

To confirm the precise insertion of constructs and expression of HSF1 proteins, genomic PCR and western blotting were performed. The genomic DNA of Pv11 cells and the clonal cell lines was extracted, then subjected to PCR. The primer sequences used in genomic PCRs are shown in Data S1 and S2. Western blotting was performed as described previously [33]. Briefly, cells were lysed with RIPA lysis buffer (Nacalai Tesque, Kyoto, Japan) for 30 min at 4 °C. After centrifugation, aliquots of the supernatant were subjected to protein quantification with a Pierce BCA Protein Assay Kit (Thermo Fisher Scientific) and 20 μg protein/lane was used for SDS-PAGE. After transferring to a PVDF membrane, the membrane was blocked with 1% skimmed milk in TBS with 0.1% Tween 20 (TBST) at 4 °C overnight. Anti-PvHSF1 antibody was generated against peptide ETMNRVLHEVKNMRGRQ in a rabbit (Merck) and used 1:2000 in 1% skimmed milk at room temperature for 1 h. After washing the membrane with TBST, secondary antibody (goat anti-rabbit IgG (H+L) HPR 65-6120, Thermo Fisher Scientific) was used 1:2000 in 1% skimmed milk at room temperature for 1 h. After washing the membrane, chemiluminescent signals from ECL Prime detection reagents (Cytiva, Little Chalfont, Buckinghamshire, UK) were captured on a ChemiDoc™ Touch imaging system (Bio-Rad, Hercules, CA, USA).

### 4.5. Desiccation-Rehydration Experiment and Calculation of Survival Rate

The procedures for desiccation and rehydration were performed as described before [15,18]. Briefly, Pv11 cells and the clonal cell lines were incubated in trehalose mixture (600 mM trehalose containing 10% (*v*/*v*) IPL-41 medium) at a density of 2 × 10^7^ cells per mL for 48 h at 25 °C. After preincubation, the cells were recovered by centrifugation and resuspended in fresh trehalose mixture. Forty microliters of the cell suspension containing 4 × 10^6^ cells were aliquoted onto a 35 mm Petri dish, and the cells were immediately transferred into a desiccator containing 1 kg of silica gel to reach a relative humidity < 10% at 25 °C. After seven to ten days, desiccated cells were rehydrated with 1 mL IPL-41 medium. One day after rehydration in IPL-41 medium, cells were stained with propidium iodide (PI; Dojindo, Kumamoto, Japan) and Hoechst 33342 (Dojindo). Then the cells were subjected to image acquisition using a BZ-X700 microscope (Keyence, Osaka, Japan) to visualize bright-field images, and PI and Hoechst fluorescence. The survival rate was calculated as the ratio of the number of live cells (Hoechst positive and PI negative) to that of total cells (Hoechst positive). 

### 4.6. HSP Gene Prediction

HSP genes in *P. vanderplanki* were predicted by the presence of HSP-related domains, including IPR013126, IPR031107, IPR008978, IPR001623, IPR002423, IPR003594, IPR001404, and blastp against Hspd1 (Hsp60), Hspe1 (Hsp10), and Hspa9 (mtHsp70) [59]. Additional filters for HSPs were based on molecular weight similarity to each group of HSPs, i.e., HSP10, HSP20, HSP40, HSP60, HSP70 and HSP90 (Data S9). The *g239*, *g240* and *g241* gene sequences were combined and analyzed together because these ‘genes’ were predicted to comprise a single gene in the newest genome assembly (version 5.2: NCBI JADBJN000000000).

### 4.7. Library Preparation for Illumina Sequencing

Total RNA in T0 and T48 samples was extracted with an RNA Plus kit (Takara Bio, Shiga, Japan) and genomic DNA was digested with TURBO DNase (Thermo Fisher Scientific), followed by NGS library preparation using a NEBNext Poly(A) mRNA Magnetic Isolation Module (New England BioLabs) and a NEBNext Ultra II RNA Library Prep Kit for Illumina (New England BioLabs). These libraries were sequenced on the Illumina HiseqX platform in the 150 bp × 2 paired-end mode at Macrogen Japan (Tokyo, Japan). Cap analysis of gene expression (CAGE) data were obtained from a previous report [47].

### 4.8. Quality Control and Trimming

For all experiments, we performed a quality control using FastQC v0.11.5 and MultiQC v1.9 software. Next, all libraries were trimmed with Trimmomatic-0.38 in SE or PE mode against adapters. Since different methods have specific features, we processed them separately.

### 4.9. CAGE Libraries Processing and Gene Model Update

Additional trimming steps were performed with fastx_trimmer (FASTX Toolkit 0.0.14), removeN and RNAdust 1.06. rRNA was predicted by RNAmmer 1.2. Trimmed reads were aligned to *P.vanderplanki* genome assembly v5.2 (NCBI JADBJN000000000) with BWA 0.7.1 and Hisat2-2.1.0. Reads with a low alignment score or PCR duplicates were removed. CAGE peaks were created using PromoterPipeline [60] python scripts, where the minimal distance between clusters was 20 bp, and expression at least 10 TPM in each sample. The highest peak in the cluster was considered to be a transcription start site (TSS) of a gene if the cluster was located within 1 kb of the original start site.

### 4.10. mRNA-Seq Library Processing and Analysis

Alignment of mRNA-seq trimmed reads was performed by Hisat2 in PE mode and sorted with samtools 1.9. Duplicated reads were marked with the Picard MarkDuplicates 1.115 tool. Finally, aligned reads were counted by htseq-count (HTSeq 0.6.0) with the “--nonunique none” option. Further normalization, differential expression, clustering, and gene ontology enrichment analysis was performed in R with edgeR and ClusterProfiler packages [61,62]. Processing and mapping results are shown in Table S8. Raw counts were converted into TPM (tags per kilobase per million) values (Data S15), and differentially expressed genes (DEGs) in the various samples (all samples: Figure S5b, and *Hsf1*^−/−^; HaloTag/BlaR vs *Hsf1*^−/−^; *Hsf1*-HaloTag/BlaR: Figure 3a) were identified by comparison between each T0 or T48 condition and accepted if the FDR was less than 0.05 (likelihood ratio test). TPM values of the genes identified as DEGs were combined as a mean of replicates and clustered using hclust function with “ward. D2” method based on Pearson correlation distances. Optimal numbers of clusters were defined using the Elbow method based on the total within sum of squares calculated for each k from 1 to 15. In Figure 5, the log2 (TPM + 1) values were calculated by applying log2 on TPM + 1 values, while the log2FC values were calculated by using edgeR.

### 4.11. Motif Enrichment Analysis

For motif analysis, the promoter regions (defined as the 1000 bp region straddling the TSS: +/− 500 bp around a known TSS or the TSS predicted by CAGE, if available) of each cluster were submitted to the Fimo and AME tools of MEME Suite 5.0.2 with default settings. The JASPAR2018 CORE motifs collection (MA0486.1, MA0486.2, MA0770.1 and MA0771.1 for the HSF binding motif) was used as a reference for the analysis. The Fimo threshold for the motif match was set as *p*-value < 0.0001. Individual matches were recorded in an annotation table. Motif enrichment analysis was done using the AME tool with case sequences and 10 k random genomic sequences as background. Optimized *p*-values from one-tailed Fisher’s exact test with Bonferroni correction were recorded and used for visualization.

### 4.12. ChIP-qPCR

ChIP assays and qPCR were performed with Simple ChIP Enzymatic Kit (Cell Signaling Technology, Danvers, MA, USA), following the manufacturer’s instructions with minor adaptions of the number of cells for each immunoprecipitation and chromatin digestion. Briefly, 6 × 10^7^ cells either prior to incubation with trehalose (T0) or after incubation with trehalose for 48 h (T48) were used for each immunoprecipitation. To crosslink the cells, formaldehyde was added to a final concentration of 1%, and cells were incubated for 10 min at room temperature. The cells were washed with ice-cold PBS twice, followed by preparation of nuclei and chromatin digestion. To digest DNA to a length of approximately 150–300 bp, 2 μL micrococcal nuclease (Cat#10011 by Cell Signaling Technology) was added and the samples were incubated for 20 min at 37 °C. After terminating digestion by addition of EDTA, lysates were sonicated using three or four pulses of 20 s each at setting 7 on a handy sonicator (UR-21P, Tomy Seiko, Tokyo, Japan). Approximately 5 μg digested chromatin samples and 0.25 μg mouse monoclonal anti-DDDDK-tag (M185-3, MBL, Tokyo, Japan) were used for each immunoprecipitation, and the samples were incubated on a rotating platform at 4 °C overnight. After purification using a spin column, qPCR was performed using the TB Green Premix Ex Taq II (Takara Bio) with specific primers (Table S9).

### 4.13. Statistical Analysis

All data were expressed as mean ± SD. Differences between two groups were examined for statistical significance using the Student *t*-test in Figure 1d. Statistical significance among more than three groups was examined by ANOVA followed by a Tukey post-hoc test (Figure 2d and Figure 4c). A *p*-value < 0.05 denoted a statistically significant difference. GraphPad Prism 8 software (GraphPad, San Diego, CA, USA) was used for the statistical analyses.

## 5. Conclusions

In conclusion, we successfully established the HSF1-knockout and -rescue cell lines using the CRIS-PITCh technique and investigated the role of HSF1 in Pv11 cells undergoing anhydrobiosis. Our results clearly show the pivotal role of HSF1 in the gene regulation required to successfully enter anhydrobiosis in Pv11 cells. Thus, our current techniques should advance understanding of the molecular mechanisms underlying anhydrobiosis. 

## Data Availability

Raw sequencing data of the mRNA-seq libraries generated in this study are available at NCBI GEO under accession number GSE171333.

## Supplementary Materials

The following are available online at https://www.mdpi.com/article/10.3390/ijms22115798/s1.
