# Supplementary material for: Genome-Wide Role of HSF1 in Transcriptional Regulation of Desiccation Tolerance in the Anhydrobiotic Cell Line, Pv11
Source: Int J Mol Sci. 2021 May 28;22(11):5798. doi: 10.3390/ijms22115798 (PMC8197945; doi:10.3390/ijms22115798)
Supplement: Supplementary file 1 [file ijms-22-05798-s001.zip › Supplementary Figures, Tables and methods.pdf]

Genome-wide role of HSF1 in transcriptional regulation of desiccation tolerance in the anhydrobiotic cell line, Pv11

Shoko Tokumoto[1†], Yugo Miyata[2†], Yugo Miyata Ruslan Deviatiiarov[3†], Takahiro G. Yamada[4], Yusuke Hiki[4], Olga Kozlova[3], Yuki Yoshida[5,6], Richard Cornette[2], Akira Funahashi[4], Elena Shagimardanova[3], Oleg Gusev[3,7], Takahiro Kikawada [1,2*]

[1]Department of Integrated Biosciences, Graduate School of Frontier Sciences, The University of Tokyo, Kashiwa 277-8562, Japan; 4914616910@edu.k.u-tokyo.ac.jp (S.T.)

[2]Division of Biotechnology, Institute of Agrobiological Sciences, National Agriculture and Food Research Organization (NARO), Tsukuba 305-0851, Japan; miyatay431@affrc.go.jp (Y.M.); cornette@affrc.go.jp (R.C.)

[3]Extreme Biology laboratory, Institute of Fundamental Medicine and Biology, Kazan Federal University, Kazan 420008, Russia; ruselusalbus@gmail.com (R.D.); olga-sphinx@yandex.ru (O.K.); rjuka@mail.ru (E.S.); gaijin.ru@gmail.com (O.G.)

[4]Department of Biosciences and Informatics, Keio University, Yokohama 223-8522, Japan; yamada@fun.bio.keio.ac.jp (T.G.Y.); hiki@fun.bio.keio.ac.jp (Y.H.); funa@bio.keio.ac.jp (A.F.)

[5]Institute for Advanced Biosciences, Keio University, Tsuruoka 997-0017, Japan; feketerigoremet@gmail.com (Y.Y.)

[6]Systems Biology Program, Graduate School of Media and Governance, Keio University, Fujisawa 252-8520, Japan

7Laboratory for Transcriptome Technology, RIKEN Center for Integrative Medical Sciences, RIKEN, Yokohama 230-0045, Japan

*Correspondence: kikawada@affrc.go.jp; Tel.: +81-298386170

†Contributed equally to this work.

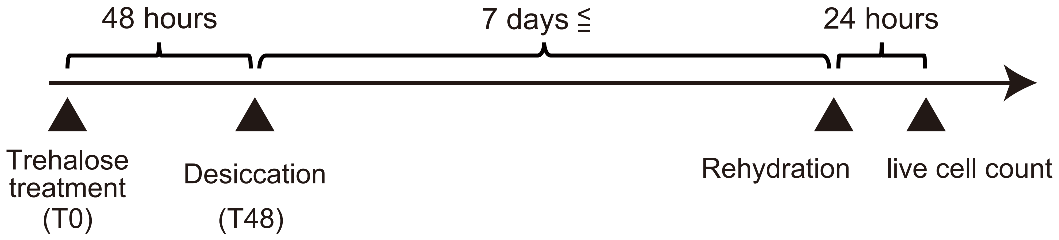

Figure S1. The scheme of trehalose treatment, desiccation and rehydration of Pv11 cells. The cells were incubated in trehalose mixture for 48 h and desiccated at least 7 days in a desiccator.

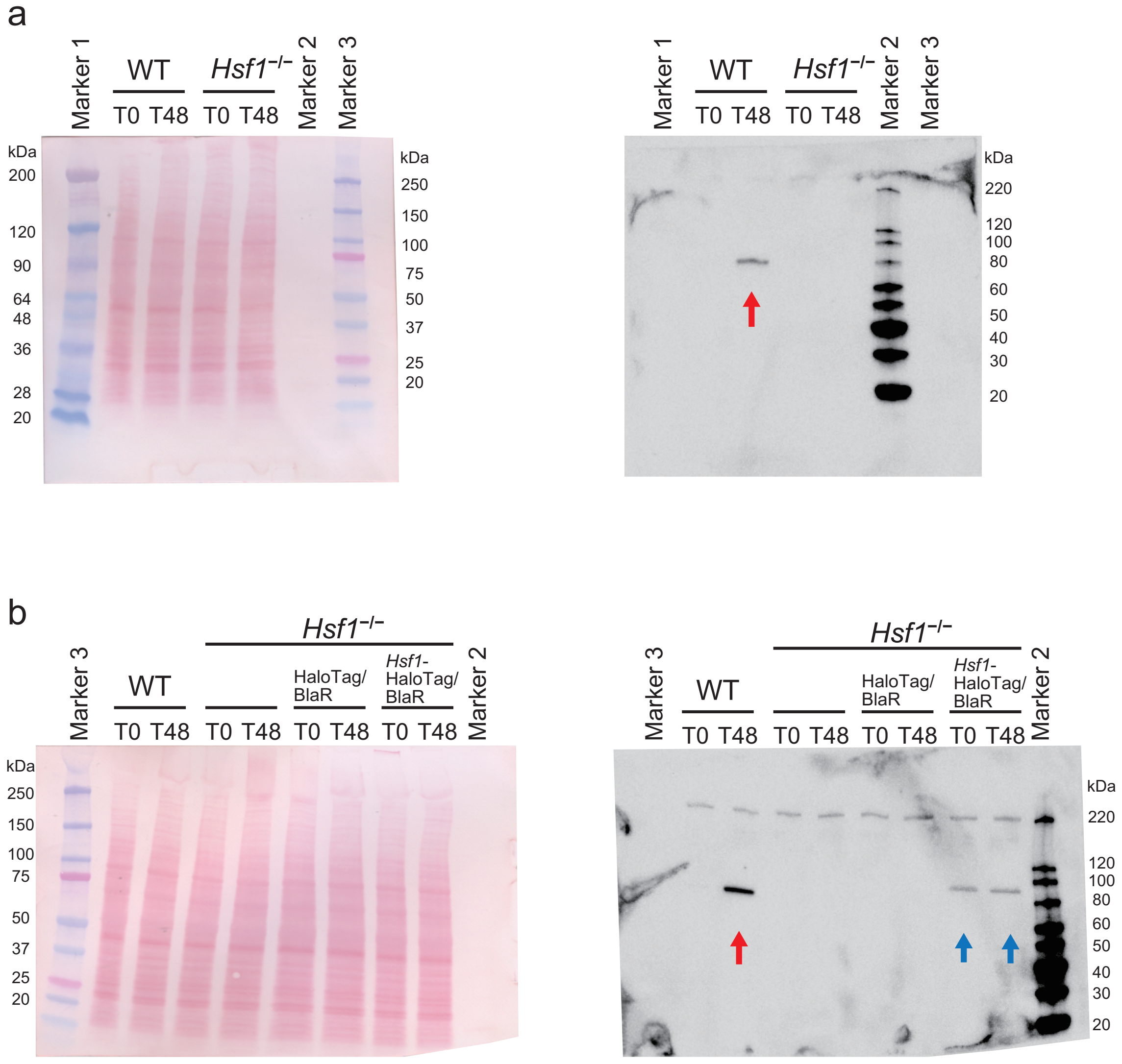

Figure S2. Validation of western blotting analysis in Figure 1c and Figure 2c. (a) Protein blotted membrane with ponceau S stain (left panel) and immune reacted membrane with anti-PvHSF1 antibody (right panel). (b) Protein blotted membrane with ponceau S stain (left panel) and immune reacted membrane with anti-PvHSF1 antibody (right panel). The molecular weight of HSF1 (red arrow) and HSF1-FLAG (blue arrow) is approximately 63 and 68 kDa, respectively, but the detected HSF1 is about 80 kDa probably due to post-translational modification [1,2]. The three types of marker were used (Marker 1, Cat#02525 by Nacarai Tesque; Marker 2, Cat#LC5602 by Thermo Fisher Scientific; Marker 3, Cat#1610374 by Bio-Rad).

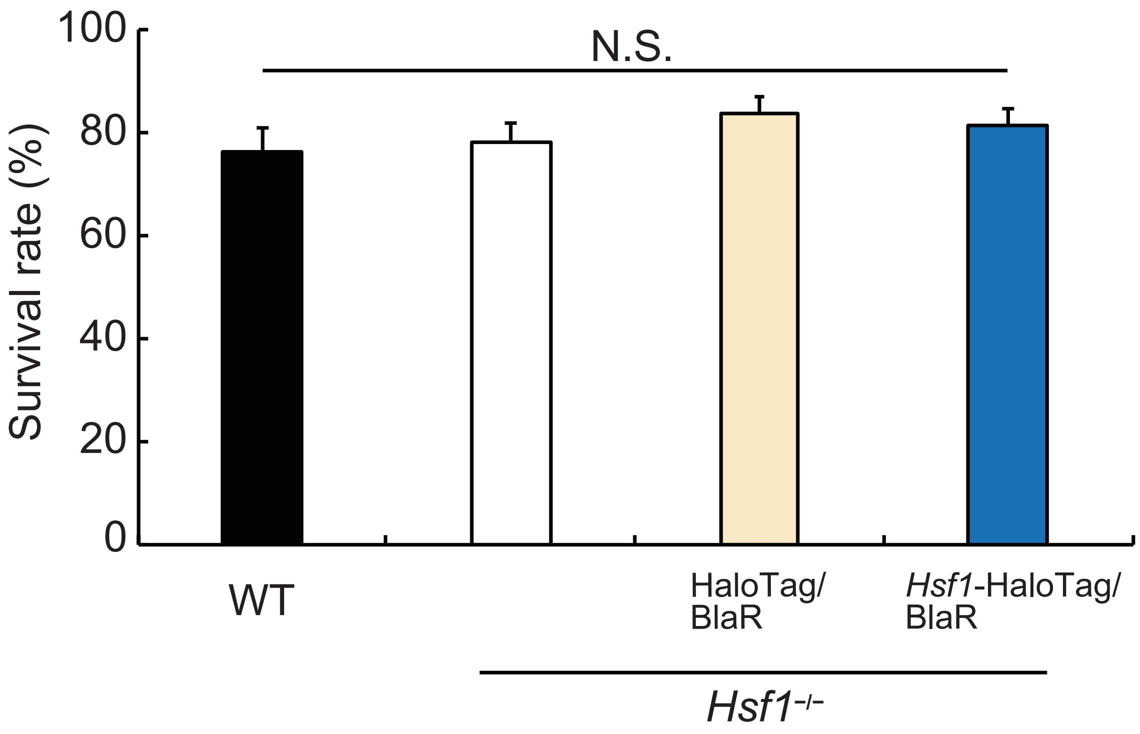

Figure S3. The survival rate of 48 hours after trehalose treatment. The cells were incubated in trehalose mixture at a density of 2 × 10^7 cells per mL for 48 h at 25°C. The survival rate was calculated as the ratio of the number of live cells (Hoechst positive and PI negative) to that of total cells (Hoechst positive). Normalized values are expressed as mean ± standard deviation (SD). N.S., not significant; n = 4 in each group.

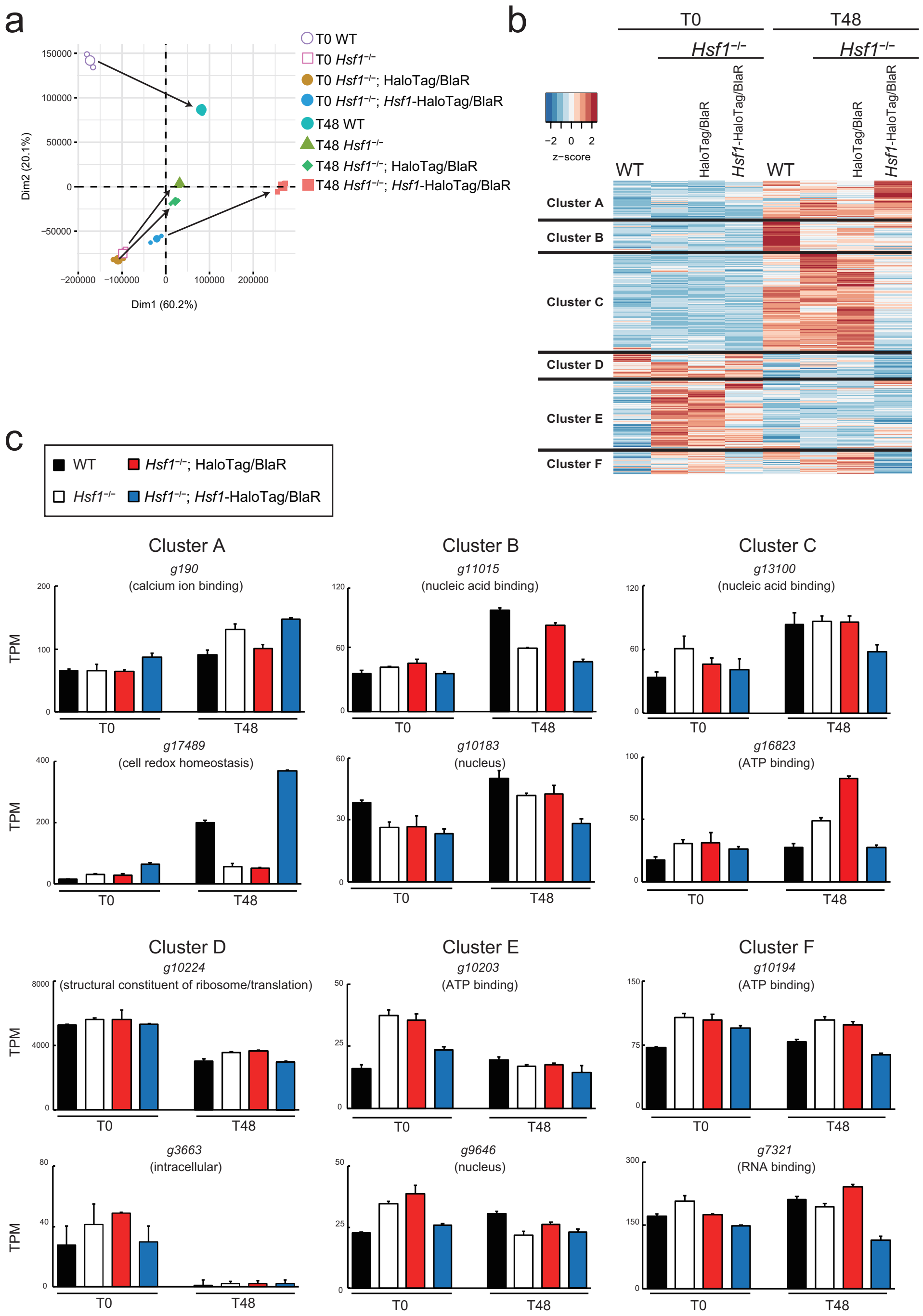

Figure S4. Comparison of the whole-genome transcription profiles of all samples. (a) PCA of all samples. (b) Hierarchical clustering based on TPM. Red and blue color indicates high expression level and low expression level, respectively. The horizontal and vertical axes show sample and the number of DEGs, respectively. (c) Examples of mRNA expression patters for each cluster in (b).

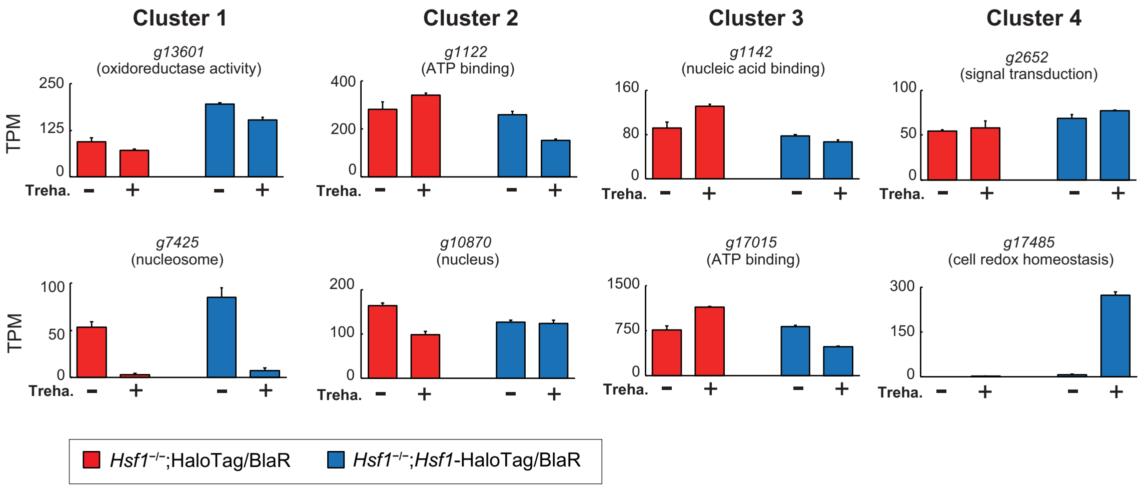

Figure S5. Examples of expression patterns in each cluster in Figure 3a. Treha. −, before trehalose treatment (T0); Treha. +, after trehalose treatment (T48).

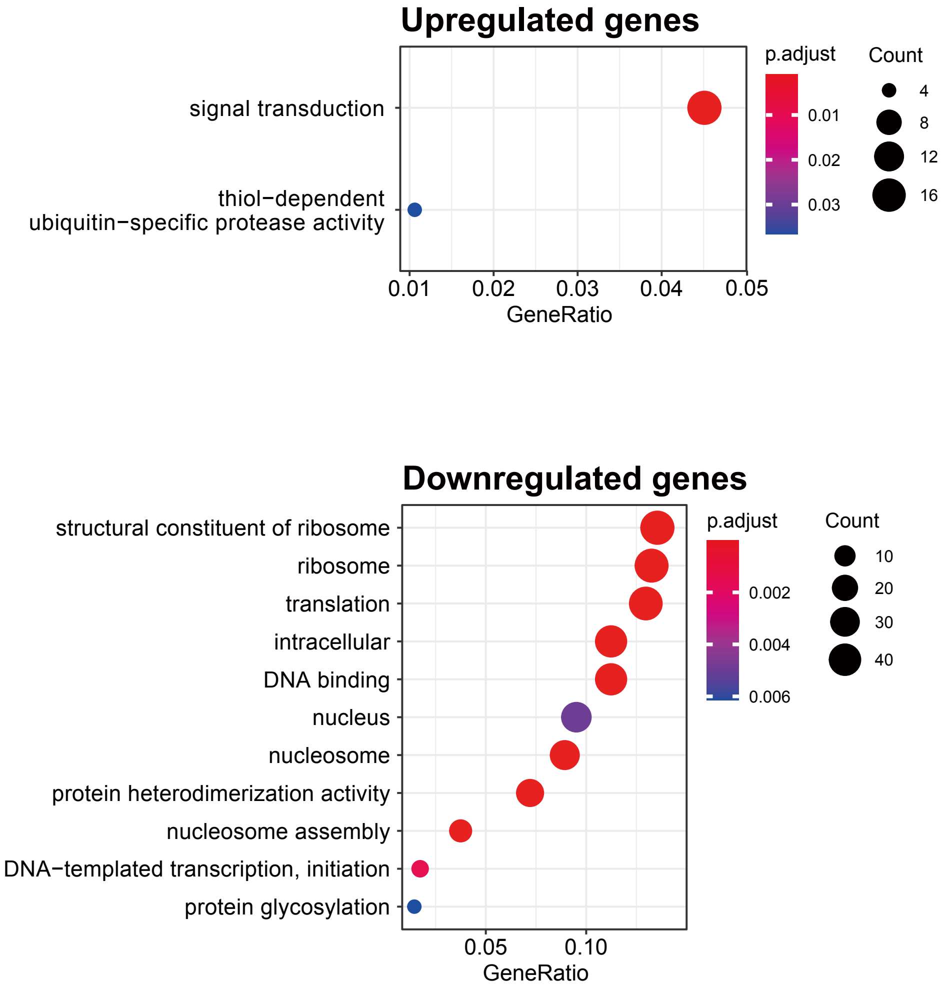

Figure S6. GO analysis of generally up- (upper panel) and downregulated (lower panel) genes in an HSF1-independent manner. All genes with or without GO annotation are shown in Data S7.

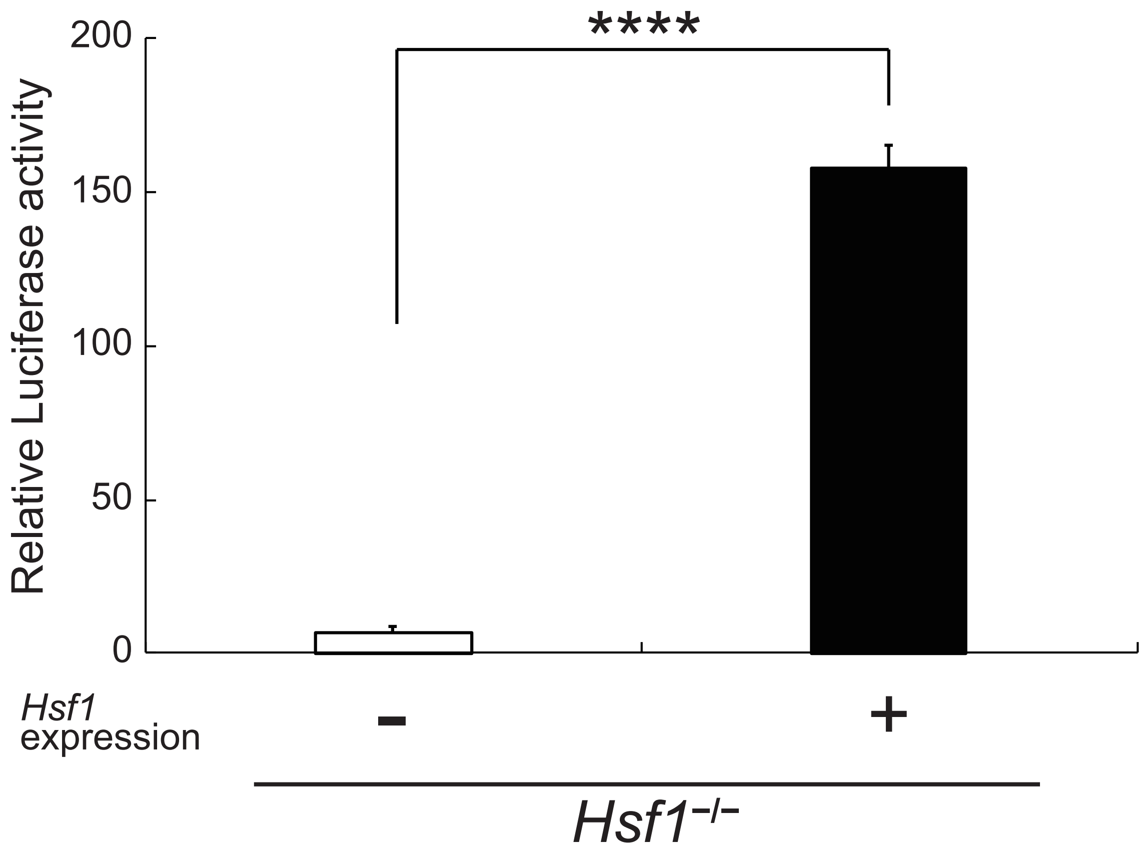

Figure S7. The transcriptional activity of HSF1 via HSE in Pv11 cells. Reporter- and internal control-vectors and either pPv121-*Hsf1* or pPv121-MCS (empty vector) were co-transfected into *Hsf1*$^{-/-}$ cells. Two days after transfection, the cells were collected and subjected to luciferase assay. Normalized values are expressed as mean ± standard deviation (SD). ****$p < 0.0001$; n = 4 in each group.

Table S1. The number of DEGs in Figure S4b.

Cluster	The number of DEGs
A	1043
B	843
C	2586
D	702
E	1817
F	645
Total	7636

Clusters were defined by hclust function with "ward. D2" method based on Pearson correlation distances.

Table S2. The number of anhydrobiosis-related genes in Figure S4b.

Gene	Cluster						
	A	B	C	D	E	F	*
Lea	23	1	0	0	0	0	3
Trx	13	2	2	0	1	0	7
Lil	13	0	0	0	0	0	0
Pimt	12	1	0	0	0	0	0
Trehalose metabolism-related	3	0	0	1	0	0	0

The genes previously reported as anhydrobiosis-related genes are picked up and counted. Lea, late embryogenesis abundant proteins; Trx, thioredoxins; Lil, lea-island-located proteins; Pimt, protein-L-isoaspartate (D-aspartate) O-methyltransferases; *, genes that are not deteced as DEGs. Trehalose metabolism-related genes include TPS, TPP, Tret1 and Treh.

Table S3. The number of DEGs in Figure 3a.

cluster number	the number of DEGs
1	1140
2	1132
3	2338
4	1320
Total	5930

Clusters were defined by hclust function with "ward. D2" method based on Pearson correlation distances.

Table S4. The number of anhydrobiosis-related genes in each cluster in Figure 3a.

Gene	Cluster				
	1	2	3	4	*
Lea	1	0	0	26	0
Trx	2	0	2	17	4
Lil	0	0	0	13	0
Pimt	0	0	0	13	1
Trehalose metabolism-related	1	0	0	3	0

The genes previously reported as anhydrobiosis-related genes are picked up and counted. Lea, late embryogenesis abundant protein; Trx, thioredoxin; Lil, lea-island-located protein; Pimt, protein-L-isoaspartate (D-aspartate) O-methyltransferase; *, genes that are not detected as DEGs.

Table S5. HSE enricment analysis.

Cluster	The number of genes in the presence or absence of HSE*		The number of total genes	Sequence similarity (adjusted *p* -value)**				Sequence similarity (-log2 (adjusted *p* -value))			
	presence	absence	Total gene	MA0486.1	MA0486.2	MA0770.1	MA0771.1	MA0486.1	MA0486.2	MA0770.1	MA0771.1
1	331	809	1140	7.37E-04	4.31E-03	1.19E-05	3.92E-06	10.40604776	7.858096415	16.3586789	17.96071491
2	298	834	1132	-	-	-	-	-	-	-	-
3	605	1733	2338	-	-	-	-	-	-	-	-
4	447	873	1320	6.79E-07	4.02E-08	5.75E-21	2.69E-13	20.49008509	24.56822926	67.23692804	41.75745906

*The number of genes that have HSE was deteted using MEME Suite Fimo. If the *p* -value was less than 0.0001, then recorded as "have HSE genes".

**Motif enrichment analysis done in MEME Suite AME. The optimal enrichment p-value of the motif according to the statistical test (Fisher's exact test), adjusted for multiple tests using a Bonferroni correction.

Table S6. Primers for vector constructions.

Primer set	PCR fragment	forward primer (5'-3')	reverse primer (3'-5')
1	AcGFP1	AAGAAAATATTTTCTGAAAAAAGGATCCCAACATGGTGAGCAAGGG	TGTGGCACCGGAACCCTTGTACAGCTCATCCATG
2	P2A-ZeoR	GCTGTACAAGGGTTCCGGTGCCACAAACTTCAGTTTGTTAAAGCAGGCCGGAGACGTCGAAGAGAACCCCGGACCGATGGCCAAGTTGACCAGTG	TAGGCTTACCTTCGAACCGCGGGCCCTCTAGATTAGTCCTG
3	gRNA#2-LμH-121-AcGFP1-P2A-ZeoR-RμH -gRNA#2	AGAGCGTGTGTGGCCCGATGGGGGGAAATTTAGGTGTCCCTGCCACGCGCTTGAAAGGAGTG	AGAGCGTGTGTGGCCCGATGGGGTCGCCATAATTTTGCCAAGACTTCAATTATGATACATGAATAAACA
4	*Hsf1* cDNA	CAGTGATCTTGAATTCATTTTAGAAAGTGAATC	GAGTTTTTCAAAAAAAATATTTTATTCATTTCTCAAAAAAAAAATTG
5	*Hsf1* -3xFLAG	AAGAAAATATTTTCTGAAAAAAGGATCCTAAAAATGCATCCGATCGAGAGTG	TAGGCTTACCTTCGAACCGCGGGATTTACTTATCGTCATCGTCTTTGTAATCAATATCATGATCCTTGTAGTCTCCGTCGTGGTCCTTATAGTCTGCATTGTTGCTAACATTATTTAGC
6	*Hsf1* -3xFLAG	GACGTCGAAGAGAACCCCGGACCGATGCATCCGATCGAGAGTG	CACCGGAACCCTTATCGTCATCGTCTTTGTAATC
7	P2A-HaloTag	TGACGATAAGGGTTCCGGTGCCACAAAC	TTAGGATGTTGTTATAAGATTTTAGCCGGAAATCTCGAGCGTC
8	BlaR	GACGTCGAAGAGAACCCCGGACCGATGGCCAAGCCTTTGTCTC	TTAGGATGTTGTTATAAGATTTTAGCCCTCCCACACATAACC
9	pPv202bp promoter	GCTAGCCTCGAGGATATCAAGATCTACAAACACGTTCAAAATCATATTTTC	CCAACAGTACCGGATTGCCAAGCTTTTTTTTCAGAAAATATTTTCTTTTGTC
10	PvGapdh promoter	GCTAGCCTCGAGGATATCAAGATCTAATATACTAAGTGTAAAAGCAATTG	CCAACAGTACCGGATTGCCAAGCTTATCGATCAAATTTCTAACTTTAG
11	*Hsf1*	AAGAAAATATTTTCTGAAAAAAggatccaaATGCATCCGATCGAGAGTG	TAGGCTTACCTTCGAACCGCGGctTTATGCATTGTTGCTAACATTATTTAGC

The primers were designed using the NEBuilder Assembly Tool Verdion1 (https://nebuilderv1.neb.com/).

Table S7. The combination of transfected donor vectors.

Established cell line	Donor Vectors	
	1	2
Hsf1 $^{-/-}$	pCR-*Hsf1* gRNAμH-121-AcGFP1-P2A-ZeoR	-
Hsf1 $^{-/-}$; HaloTag/BlaR	pCR4-*g7775* gRNAμH-P2A-HaloTag	pCR4-*g7775* gRNAμH-P2A-BlaR
Hsf1 $^{-/-}$; *Hsf1* -HaloTag/BlaR	pCR4-*g7775* gRNAμH-P2A-*Hsf*-3xFLAG-P2A-HaloTag	pCR4-*g7775* gRNAμH-P2A-BlaR

These donor vectors were transfected at a concentration of 0.03-0.1 pmol. The pCR4-*g7775* gRNAμH-P2A-BlaR was previously constructed in our study.

Table S8. Processing and mapping.

Sample	Picard: deduplication stats						HTSeq Count			
	Unique Pairs	Unique Unpaired	Duplicate Pairs Optical	Duplicate Pairs Nonoptical	Duplicate Unpaired	Unmapped	Assigned	Ambiguous	Alignment Not Unique	No Feature
WT_T0-1	17744216	342796	8315886	2359710	2769595	7099497	13581193	67607	2454183	16791903
WT_T0-2	16500192	342324	6917798	2060926	2499355	6102539	12203353	58993	2088677	15098654
WT_T0-3	15312082	331501	6406878	1922688	2387537	5908090	11331144	56747	1961295	14067660
WT_T48-1	21014452	213900	7130992	2224304	1391125	3772231	13485192	77778	2666206	17308323
WT_T48-2	16417312	357996	5141460	1649528	2096162	4450720	10857269	63133	2055070	13780777
WT_T48-3	18687444	372268	6003902	1932704	2373327	5105643	12369050	69711	2495303	15814718
Hsf1 $^{-/-}$_T0-1	11566222	283256	3694462	1199564	1482908	4073576	7840554	31605	1554338	9620138
Hsf1 $^{-/-}$_T0-2	16475896	324917	6651050	1959260	2256786	5953237	11803954	44565	2416198	14756602
Hsf1 $^{-/-}$_T0-3	14369316	295517	5016442	1578746	1780813	4838876	9874913	37053	1818693	12281064
Hsf1 $^{-/-}$_T48-1	15977990	359528	4942972	1578632	2135979	4538837	10767831	56099	1746029	13327710
Hsf1 $^{-/-}$_T48-2	15592832	367219	4769592	1508048	2125092	4468649	10445405	57272	1681196	13036487
Hsf1 $^{-/-}$_T48-3	16642358	351026	5010360	1629448	2086748	4484900	11000242	56393	1802682	13799185
Hsf1 $^{-/-}$; HaloTag/BlaR_T0-1	18724702	345213	7428008	2245278	2579265	6942892	13394818	51410	2634959	16732763
Hsf1 $^{-/-}$; HaloTag/BlaR_T0-2	16580826	349540	7021872	2076316	2578817	7168081	12302148	44152	2604377	15141072
Hsf1 $^{-/-}$; HaloTag/BlaR_T0-3	11985794	91953	3336414	1122744	464592	2718931	7221398	24164	1451290	9193863
Hsf1 $^{-/-}$; HaloTag/BlaR_T48-1	13982080	306981	4033148	1295468	1652659	3805156	9068587	43179	1630689	11377734
Hsf1 $^{-/-}$; HaloTag/BlaR_T48-2	15287954	358049	4507072	1475838	2056979	4372218	10002489	51488	1891966	12749146
Hsf1 $^{-/-}$; HaloTag/BlaR_T48-3	15994514	454301	4851628	1550136	2686613	5370326	10713183	54528	2279123	13730426
Hsf1 $^{-/-}$; *Hsf1* -HaloTag/BlaR_T0-1	20893958	364191	8914736	2722020	3085420	8832121	15355239	75142	3305042	19168520
Hsf1 $^{-/-}$; *Hsf1* -HaloTag/BlaR_T0-2	12509068	277047	4310538	1367584	1650998	4973597	8471134	43691	1846041	10769287
Hsf1 $^{-/-}$; *Hsf1* -HaloTag/BlaR_T0-3	13211692	292794	4616150	1459502	1802256	5228012	9058347	44689	1803089	11463973
Hsf1 $^{-/-}$; *Hsf1* -HaloTag/BlaR_T48-1	13924608	351484	4626958	1430084	2142129	4838909	9449334	71880	1819781	12086436
Hsf1 $^{-/-}$; *Hsf1* -HaloTag/BlaR_T48-2	20366672	391239	7783968	2370782	3080089	6871950	14486439	103533	2658170	18210162
Hsf1 $^{-/-}$; *Hsf1* -HaloTag/BlaR_T48-3	15107630	317984	5027232	1579068	2000832	4712646	10202281	72470	1859158	12879622

Unique Pairs: the number of unique read pairs

Unique Unpaired:the number of unique single reads

Duplicate Pairs Optical: the number of cases when single cluster has falsely been called as two by Illumina softweare

Duplicate Pairs Nonoptical: the number of duplicates in nearby wells, PCR, and sister strand duplicates

Duplicate Unpaired: the number of unpaired duplicates

Unmapped: the number of unmapped reads

Assigned: the number of unique reads assigned to single feature (gene)

Ambiguous: the number of reads matched to more than one features

Alignment Not Unique: the number of not uniquely aligned reads

No Feature: the number of reads without any assigned features

Table S9. Primers for ChIP-qPCR in Figure 4c.

Target	forward primer (5'-3')	reverse primer (3'-5')
g16187 promoter	TCCTGAGGCATCTCTAACACC	CTGCACAAACTGCTCAGGAA
g16356 promoter	TTTTTCCGGCAAATTTTTCT	TGGATCATTGATGCGACAGT
g17488 promoter	CGGTCTGGGATTTTTCTTTTC	TTTTCTCGAATATTGCTTAATTTTT
g5646 promoter	TTTTTCTTGCGCTTTTGAAAT	TTCTCGAATGTTCCATTTTGC

The primers were designed using the Primer3Plus (http://www.bioinformatics.nl/cgi-bin/primer3plus/primer3plus.cgi).

Supplementary materials and methods

Vector construction

To construct the p-3×HSE-Pv202bp-Nluc reporter vector, first, pPv202bp-Nluc was constructed. The Pv202bp minimal promoter [3] was cloned and amplified using specific primers (Table S6: set 9) from pTetO-202bp-AcGFP1 [3] and inserted between HindIII and BglII sites of pNL1.1 (Promega, Fitchburg, WI) using NEBuilder HiFi DNA Assembly Mater Mix. To insert the tandemly repeated HSE sequence (MA0486.1, http://jaspar.genereg.net/) into the pPv202bp-Nluc, the vector was digested with BglII and EcoRV and the digested small fragment was replaced with the HSE sequence generated by annealing the following oligonucleotides: sense: 5'-ATCcTTCtaGAAacTTCtcTTCtaGAAacTTCtcTTCtaGAAacTTCtA-3'; antisense: 5'-GATCTaGAAgtTTCtaGAAgaGAAgtTTCtaGAAgaGAAgtTTCtaGAAgGAT-3' (p-3×HSE-Pv202bp-Nluc; Data S16). For construction of the luc2-reference vector, the *PvGapdh* promoter was amplified from pPGK vector [4] by PCR (Table S6: set 10) and inserted between HindIII and BglII sites of pGL4.10 (Promega) using NEBuilder HiFi DNA Assembly Mater Mix (pPvGapdh-luc2: Data S17). For construction of pPv121-*Hsf1* expression vector, *Hsf1* ORF was amplified with specific primers (Table S6: set 11) from cDNA of dried *P. vanderplanki* larvae and inserted pPv121-MCS [3] digested with BamHI and SacII using NEBuilder HiFi DNA Assembly Mater Mix (pPv121-*Hsf1*: Data S18).

Transfection and luciferase assay

The *Hsf1*$^{-/-}$ cells were seeded at a density of 3×10^5 cells per mL into a 25 cm^2 cell culture flask and grown at 25°C for 4-6 days before transfection. Transfection into Pv11 cells was carried out using a NEPA21 Super Electroporator (Nepa Gene). A mixture of 0.5 µg of either pPv121-*Hsf1* or pPv121-MCS, 2 µg p-3×HSE-Pv202bp-Nluc reporter vector and 5 µg PvGapdh-luc2 vector was transfected into the cells. Two days after transfection, the cells were collected, and luciferase activity was measured using an ARVO luminometer (PerkinElmer, Waltham, MA) with the Nano-Glo Dual-Luciferase Reporter Assay System (Promega).

HSF1-independent genes selection

To obtain genes that are generally up- and downregulated by HSF1-independent manner during trehalose treatment in Pv11 cells, no-DE genes (FDR > 0.05) were analyzed. FDR and log2FC values were calculated by comparison of TPM of *Hsf1*$^{-/-}$; HaloTag/BlaR between T0 vs T48 and of *Hsf1*$^{-/-}$; *Hsf1*-HaloTag/BlaR between T0 vs T48, then genes were extracted if the FDR is less than 0.05 in both

cells. Up- and downregulated genes were determined with log2FC values: log2FC >0, upregulated genes; log2FC <0, downregulated genes (Data S6).

References

1. Hietakangas, V.; Ahlskog, J.K.; Jakobsson, A.M.; Hellesuo, M.; Sahlberg, N.M.; Holmberg, C.I.; Mikhailov, A.; Palvimo, J.J.; Pirkkala, L.; Sistonen, L. Phosphorylation of serine 303 is a prerequisite for the stress-inducible SUMO modification of heat shock factor 1. *Mol Cell Biol* **2003**, *23*, 2953-2968, doi:10.1128/mcb.23.8.2953-2968.2003.
2. Xu, Y.M.; Huang, D.Y.; Chiu, J.F.; Lau, A.T. Post-translational modification of human heat shock factors and their functions: a recent update by proteomic approach. *J Proteome Res* **2012**, *11*, 2625-2634, doi:10.1021/pr201151a.
3. Tokumoto, S.; Miyata, Y.; Usui, K.; Deviatiiarov, R.; Ohkawa, T.; Kondratieva, S.; Shagimardanova, E.; Gusev, O.; Cornette, R.; Itoh, M., et al. Development of a Tet-On Inducible Expression System for the Anhydrobiotic Cell Line, Pv11. *Insects* **2020**, *11*, doi:10.3390/insects11110781.
4. Sogame, Y.; Okada, J.; Kikuta, S.; Miyata, Y.; Cornette, R.; Gusev, O.; Kikawada, T. Establishment of gene transfer and gene silencing methods in a desiccation-tolerant cell line, Pv11. *Extremophiles* **2017**, *21*, 65–72, doi:10.1007/s00792-016-0880-4.
